# Discovery of a novel Mediterranean *Haemaphysalis* (*Ornithophysalis*) *doenitzi* group tick species infesting *Falco eleonorae* on Antikythira Island, Greece

**DOI:** 10.1017/S0031182024000866

**Published:** 2024-08

**Authors:** Lidia Chitimia-Dobler, Christos Barboutis, Anastasios Bounas, Christina Kassara, Ben J. Mans, Anastasios Saratsis

**Affiliations:** 1 Bundeswehr Institute of Microbiology, Munich, Germany; 2 Fraunhofer Institute of Immunology, Infection and Pandemic Research, Penzberg, Germany; 3 Antikythira Bird Observatory, Hellenic Ornithological Society/BirdLife Greece, Athens, Greece; 4 Epidemiology, Parasites and Vectors, Agricultural Research Council-Onderstepoort Veterinary Research, Onderstepoort, South Africa; 5 Department of Life and Consumer Sciences, University of South Africa, Pretoria, South Africa; 6 Department of Zoology and Entomology, University of the Free State, Bloemfontein, South Africa; 7 Veterinary Research Institute, Hellenic Agricultural Organisation-Dimitra, Thermi, Greece

**Keywords:** 16S, Aegean Sea, COI, Falconidae, Greece, *Haemaphysalis*, Ixodidae, mitochondrial genome, NGS, tick

## Abstract

Eleonora's falcon (*Falco eleonorae* Géné, 1839) is a well-known long-distance migrant of the Afro-Palaearctic flyway, a summer breeder of the Mediterranean region and North-west Africa and a winter resident of Madagascar and surrounding areas, thus characterized as a double endemic. Within the context of a long-term monitoring and conservation programme on Antikythira Island, Greece, which accommodates one of the largest concentrations of breeding pairs of Eleonora's falcons globally, birds were subjected to regular inspections for the presence of ticks from 2017 to 2023. In total, 104 adults and 149 nymphs (all belonging to *Haemaphysalis* genus) were collected. All ticks, apart from 2 nymphs, exhibited broadly salient palpi and did not possess the pronounced palpal segment 2 spurs or spur-like angles that are characteristic of adults, nymphs and most larvae of *Rhipistoma*, thus placed them in the *Ornithophysalis* subgenus. Following comprehensive morphological assessment and genetic analysis of the mitochondrial genome by means of next-generation sequencing of both adult and nymphal stages of the ticks, our empirical findings substantiate the delineation of a previously unclassified species. This taxonomic assignment situates the newly described species within the *Ornithophysalis* subgenus and the *Haemaphysalis doenitzi* group, marking its presence for the first time within the Western Palaearctic region.

## Graphical abstract

**Figure:**
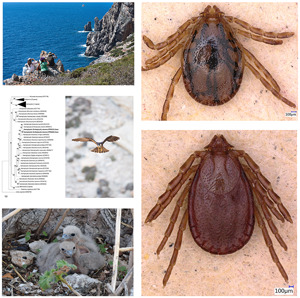

## Introduction

In the family Ixodidae, there are currently 762 recognized species, with the *Haemaphysalis* Koch, 1844, genus comprising 176 of them (Guglielmone *et al*., 2023). Within the Western Palaearctic, so far, at least 11 out of the 18 endemic *Haemaphysalis* species with a Palaearctic distribution have been recognized: *Haemaphysalis* (*Rhipistoma*) *adleri* Feldman-Muhsam, 1951, *Haemaphysalis* (*Rhipistoma*) *caucasica* Olenev, 1928, *Haemaphysalis* (*Haemaphysalis*) *concinna* Koch, 1844, *Haemaphysalis* (*Rhipistoma*) *erinacei* Pavesi, 1884, *Haemaphysalis* (*Rhipistoma*) *hispanica* Gil Collado, 1938, *Haemaphysalis* (*Alloceraea*) *inermis* Birula, 1895, *Haemaphysalis* (*Allophysalis*) *kopetdaghica* Kerbabaev, 1962, *Haemaphysalis* (*Rhipistoma*) *leachi* Audouin, 1826, *Haemaphysalis* (*Segalia*) *parva* Neumann, 1897, *Haemaphysalis* (*Aboimisalis*) *punctata* Canestrini and Fanzago, 1878 and *Haemaphysalis* (*Herpetobia*) *sulcata* Canestrini and Fanzago, 1878 (Estrada-Peña *et al*., 2017; Guglielmone *et al*., 2023). With some exceptions (Hornak *et al*., 2016*a*, 2016*b*; Kelava *et al*., 2023), the phylogenetic relationships of the *Haemaphysalis* ticks in this zoogeographic region have gained little attention, despite their economic, medical and veterinary importance.

The subgenus *Ornithophysalis* Hoogstraal and Wassef, 1973, defined within the *Haemaphysalis* genus, comprises 19 known species (Hoogstraal and Wassef, 1973; Saito *et al*., 1974; Camicas *et al*., 1998) that serve as the main phylogenetic branch for the emergence of more than 30 species, primarily parasitizing mammals, within the subgenus *Rhipistoma* Koch, 1844 (Hoogstraal *et al*., 1965; Estrada-Peña, 1990; Apanaskevich and Tomlinson, 2019). *Ornithophysalis* species predominantly exhibit an affinity for avian hosts, although there is a subset that displays dual parasitism on both avian and mammalian hosts, while some exclusively infest mammals (Hoogstraal and Wassef, 1973). The subgenus can be taxonomically categorized into either 4 or 5 distinct structural-biological groups of species, given uncertainties on *Haemaphysalis verticalis* Itagaki, Noda and Yamaguchi, 1944 status, each exhibiting specific host preferences and geographical distribution (Emel'yanova and Hoogstraal, 1973; Hoogstraal and Wassef, 1973; Camicas *et al*., 1998). The taxon known as the *Haemaphysalis* (*Ornithophysalis*) *doenitzi* group, as defined by Hoogstraal and Wassef (1973), encompasses multiple species with distinct geographical distributions. Within this group, the species *H. doenitzi* Warburton and Nuttall, 1909 is observed in the Australasian (Australia, Papua New Guinea), Oriental (south China, India, Laos, Malaysia, Myanmar, south and central Nepal, the Philippines, Singapore, Sri Lanka, Taiwan, Thailand, Vietnam) and Eastern Palaearctic (only in north China) faunal regions. The species *Haemaphysalis phasiana* Saito, Hoogstraal and Wassef, 1974 is identified in the Oriental (south China) and Eastern Palaearctic (Japan, South Korea, Turkmenistan, Russia) regions, *Haemaphysalis madagascariensis*, as described by Colas-Belcour and Millot (1948), in the Afrotropical region (only Madagascar) and finally, the Afrotropical region (30 countries) hosts the species *Haemaphysalis hoodi* Warburton and Nuttall, 1909 (Hoogstraal and Wassef, 1973; Saito *et al*., 1974; Guglielmone *et al*., 2023). *Haemaphysalis pavlovskyi* Pospelova-Shtrom, 1935 is considered a valid tick species that can be found in the Palaearctic region (Kyrgyzstan, Tajikistan) according to some authors (Filippova, 1997; Guglielmone *et al*., 2009, 2014, 2020), whereas others considered it a synonym of *H. doenitzi* (Camicas *et al*., 1998; Kolonin, 2009). Fifty years ago, Hoogstraal and Wassef (1973) noted that *Ornithophysalis* populations in bordering and adjacent ecological and geographical areas of the Palaearctic and Oriental faunal regions, including the Western Palaearctic, were in special need of more investigation given the lack of data.

The Western Palaearctic hosts the largest avian migration system where billions of land birds travel twice each year between their breeding and wintering grounds (Hahn *et al*., 2009). A well-known long-distance migrant of the Afro-Palaearctic flyway is Eleonora's falcon (*Falco eleonorae* Géné, 1839), a summer breeder of the Mediterranean region and North-west Africa and a winter resident of Madagascar and surrounding areas (Walter, 1979), thus characterized as a double endemic (Bildstein, 2006).

Little is known regarding ectoparasitic infestations and related pathogens transmitted by them to Eleonora's falcons (Wink *et al*., 1979; Gutiérrez-López *et al*., 2015; Gangoso *et al*., 2019; Laid *et al*., 2019). Regarding ticks, past studies from breeding colonies situated at Aegean Sea islets (Dionysades islets north-east of Crete) in Greece highlighted the infestation of both adult and juvenile birds by adult and immature *Haemaphysalis numidiana* Neumann, 1905 (Wink *et al*., 1979), which is a synonym of *Haemaphysalis erinacei* Pavesi, 1884 (Guglielmone *et al*., 2014, 2020). However, it is important to note that in a later publication summarizing the above findings this species was erroneously referred to as *Haemaphysalis hoody* (Wink and Ristow, 2000), a designation that does not correspond to a valid tick species and was probably a typographical error, with the intended name being *H. hoodi*.

Within the context of a long-term monitoring and conservation programme on Antikythira Island, Greece, which accommodates one of the largest concentrations of breeding pairs of Eleonora's falcons globally (Dimalexis *et al*., 2008; Kassara *et al*., 2019), birds were subjected to regular inspections for the presence of ticks. The current study describes a new species using morphological and molecular criteria to differentiate it from closely related species in the *H. doenitzi* group.

## Materials and methods

### Study site

The study was centred on Antikythira Island, located in the southern Aegean Sea, Greece (35.864N, 23.309E). Antikythira is a relatively hilly island, positioned between Kythira and Crete, with a maximum altitude of nearly 400 m above sea level and covering an area of around 20 km^2^. The island is predominantly covered with phrygana and maquis vegetation, whereas agricultural activities are currently limited to a small portion of the island (Kassara *et al*., 2019). Apart from a few scattered cultivated trees, such as olive and almond trees, the maquis vegetation on the island remains relatively low due to the grazing activities of goats (Kassara *et al*., 2019). The presence of ticks infesting other long-distance migratory birds was recorded in Antikythira, including passerines and near-passerines, given its importance as an important refuelling site (Barboutis *et al*., 2022). Among these ticks, the most prevalent species identified were *Hyalomma marginatum* and *Hyalomma rufipes* (Wallménius *et al*., 2014).

### Field survey

Field surveys were undertaken over a period of approximately 5 days each in May and September of the respective year. In 2017 and 2018, ticks opportunistically observed on falcons were collected. From May 2019 to September 2023, a 5 min comprehensive examination for ticks was systematically conducted on virtually all handled falcons (Keskin *et al*., 2021). The capture of these falcons was carried out using mist nets in proximity to a natural water pond complex, as part of an ongoing long-term monitoring project in the region run by the Hellenic Ornithological Society. In September, predominantly female Eleonora's falcons were captured, most likely nesting on Antikythira and/or nearby islets (Kassara *et al*., 2023). In contrast, during May, the captured individuals were predominantly males that had recently arrived in the Mediterranean and presumably engaged in nest-site selection in the vicinity of the study area for the upcoming breeding period (Kassara *et al*., 2023). Additionally, a few nestlings were checked (*n* = 11, of which 8 siblings, pertaining to 6 nests of which 1 was revisited in subsequent years). Ticks were exclusively found on the head, and were collected and stored in absolute ethanol.

### Morphological identification

Ticks were first identified using morphological keys (Warburton and Nuttall, 1909; Nuttal and Warburton, 1915; Hoogstraal, 1956; Hoogstraal and Wassef, 1973; Saito *et al*., 1974; Horak *et al*., 2018), under a Keyence VHX-900F microscope (Itasca, IL, USA). Specimens from all found life stages (females, males and nymphs) were photographed and measured for the morphological description using the same microscope.

### 16S sequencing

DNA was extracted from 1 female (collected on 20 May 2021), 2 males (both collected on 14 May 2022) and a nymph (collection date: 21 September 2019), previously used for both morphological descriptions and provided photos in the new species description section, using the QIAamp Mini DNA extraction kit (Qiagen, Hilden, Germany) according to the manufacturer's instructions. The 16S rRNA gene of ticks was amplified according to Halos *et al*. *(*2004), visualized in 1.5% agarose gel, purified using a QIAquick^®^ PCR Purification Kit (Qiagen, Hilden, Germany), and subsequently bi-directionally sequenced, consensus sequences derived and submitted to GenBank (PP769621–PP769624).

### Next-generation sequencing, assembly and mitochondrial genome annotation

Genomic DNA was extracted from 2 female ticks (collected on 14 May 2022 and 17 May 2022, respectively) using the QIAamp DNA Blood Mini Kit (Qiagen), processed using the MGIEasy Universal DNA Library Prep kit (MGI, Shenzhen, China) and sequenced on the MGI DNBSEQ-G400 sequencing instrument using the PE150 (paired-end 2 × 150 bp) format (Agricultural Research Council-Biotechnology Platform, South Africa). Paired-end sequence data were quality trimmed (0.001 quality limit) and MGI adapters removed using CLC Genomics Workbench v.20.1 software (Qiagen). Standard assembly parameters (mismatch cost-2, insertion cost-3, deletion cost-3, length fraction-0.9, similarity-0.9, minimum contig length-200 and automatic bubble size) were used and assembly performed using a word size of 49 in CLC Genomics Workbench v.20.0 software (Qiagen). Contigs were identified as mitochondrial, 18S or 28S rRNA using BLAST analysis. Final contigs were obtained by mapping data back to the contigs using CLC Genomics Workbench v.20.1 (mismatch cost-2, insertion cost-3, deletion cost-3, length fraction-0.5 and similarity-0.9) to obtain consensus sequences and final coverage values (coverage 1283 and 6054). The mitochondrial genome was annotated using the MITOS and ARWEN servers to identify tRNA genes (Laslett and Canbäck, 2008; Bernt *et al*., 2013). Protein coding and rRNA genes were identified using BLAST analysis (Altschul *et al*., 1990). Mitochondrial genomes have been submitted to GenBank (accession numbers: PP059219 and PP059220).

### Phylogenetic analysis

#### 16S rRNA phylogenetic analysis

Sequences from GenBank were downloaded using BLASTN analysis (Altschul *et al*., 1990) of the new species to retrieve all *Haemaphysalis* sequences and produce a non-redundant dataset with sequences that represented unique species. This yielded a final dataset of 54 sequences that was aligned using MAFFT taking rRNA secondary structure into account (Q-INS-i) (1PAM/k = 2 scoring matrix) (Katoh and Standley, 2013). Maximum-likelihood analysis was performed using IQ-Tree2 v 2.2.0 (Minh *et al*., 2020) with an alignment size of 297 bp. The most optimal substitution model used was K3Pu + F + I + G4. Nodal support was estimated using ultrafast bootstrap (*n* = 10 000) and the 50% consensus tree was reported.

#### Cytochrome oxidase I phylogenetic analysis

Sequences from GenBank were downloaded using BLASTN analysis (Altschul *et al*., 1990) of the new species to retrieve all Haemaphysalis sequences and produce a non-redundant dataset with sequences that represented unique species. As outgroup the sequence for *I. scapularis* was included. This yielded a final dataset of 26 sequences that was aligned using MAFFT taking rRNA secondary structure into account (Q-INS-i) (1PAM/k = 2 scoring matrix) (Katoh and Standley, 2013). Maximum-likelihood analysis was performed using IQ-Tree2 v 2.2.0 (Minh *et al*., 2020) with an alignment size of 636 bp. The most optimal substitution model used was TIM2 + F + I + I + R3. Nodal support was estimated using ultrafast bootstrap (*n* = 100 000) and the 50% consensus tree was reported.

#### Mitochondrial genome phylogenetic analysis

Translated protein sequences for the 13 protein genes were used for phylogenetic analysis. Multiple sequence alignments for each protein were performed separately using MAFFT with iterative alignment (FFT-NS-i) and the BLOSUM62 amino acid scoring matrix (Katoh and Standley, 2013). Maximum-likelihood analysis was performed in IQ-Tree2 v 2.2.0 (Minh *et al*., 2020). An optimal substitution model was calculated for each protein partition: ATP6 (mtInv + I + I + R5), ATP8 (mtMAM + F + G4), COI (mtMet + R4), Cytb (mtInv + I + I + R5), ND1 (mtInv + I + G4), ND2 (mtMet + F + I + I + R5), ND3 (mtInv + I + G4), ND4 (mtInv + I + G4), ND4L (mtART + G4), ND5 (mtMet + F + I + I + R6) and ND6 (mtInv + I + G4). Absent protein genes were treated as missing data. An edge-proportional partition model with proportional branch lengths (-spp) was used to allow each partition its own specific rate to accommodate different evolutionary rates between partitions. Nodal support was estimated using ultrafast bootstrap (*n* = 1 000 000) and the 50% consensus tree was reported.

## Results

A total of 110 Eleonora's falcons, comprising both adults and subadults (the latter being non-reproductively mature) were captured utilizing mist nets. Of these individuals 25 were tick infested, constituting 22.7% (95% confidence interval (CI): 15.3–31.7) of the overall sample, with 52% (13/25) of them captured during the breeding period of different years. In addition, our survey encompassed 11 nestlings, with 7 of them exhibiting tick infestations during different years, corresponding to an infestation rate of 63.6% (95% CI: 30.8–89.1) within this specific cohort. In total, 104 adults and 149 nymphs (all belonging to *Haemaphysalis* genus) were collected. All ticks, apart from 2 nymphs, exhibit broadly salient palpi and do not possess the pronounced palpal segment 2 spurs or spur-like angles that are characteristic of adults, nymphs and most larvae of *Rhipistoma*, thus placed them in the *Ornithophysalis* subgenus.

Below we describe the *Haemaphysalis* sp. nov. collected from Eleonora's falcons in this study.

### Systematic for the Ixodidae

#### Class Arachnida Lamarck, 1801 Order Parasitiformes Reuter, 1909 Suborder Ixodida Leach, 1815 Family Ixodidae Murray, 1877 *Haemaphysalis* Koch, 1844 *Haemaphysalis eleonorae* sp. nov., Chitimia-Dobler, Mans and Saratsis

Diagnostic: rectangular basis capituli, palpal segment II without spur, palpal segment III with a short ventral spur, dental formula 4/4 for adults and 3/3 for nymph, 11 festoons, with 2 enclosed by the marginal groove in females and with 1 in males.

Type host: *Falco eleonorae*

Type locality: Antikythira, Greece

Type material: Holotype male, from *Falco eleonorae*, Antikythira, Greece (35°51′36.1″N, 23°17′15.1″E), collected on 14 May 2022 by C. Barboutis; deposited in the Natural History Museum of Crete (NHMC.81.4.19216.1 ). Allotype female, with the same collection data as holotype; deposited in the NHMC (NHMC.81.4.19216.2). Paratype nymphs (3 specimens; NHMC.81.4.19217.1-3) and males (2 specimens; NHMC.81.4.19216.3-4), with the same collection data as holotype except for the collection date in the case of the nymphs (20 September 2019); deposited in the NHMC. One paratype female (ZMB_Arach 55352; collection date: 17 May 2022), one paratype male (ZMB_Arach 55353; collection date: 14 May 2022) and one paratype nymph (ZMB_Arach 55354; collection date: 21 September 2019) were deposited in the Natural History Museum of Berlin.Three paratype males with the same collection data as holotype were submitted to the National Tick Collection Onderstepoort Veterinary Institute.

Etymology: The species' name is derived from its host species, *F. eleonorae*.

ZooBank registration: The Life Science Identifier (LSID) for *Haemaphysalis eleonorae* is urn:lsid:zoobank.org:pub:6553B708-7D27-4C3F-B94A-E94E8F8F3C91.

### Description of male

**Figure 1.**
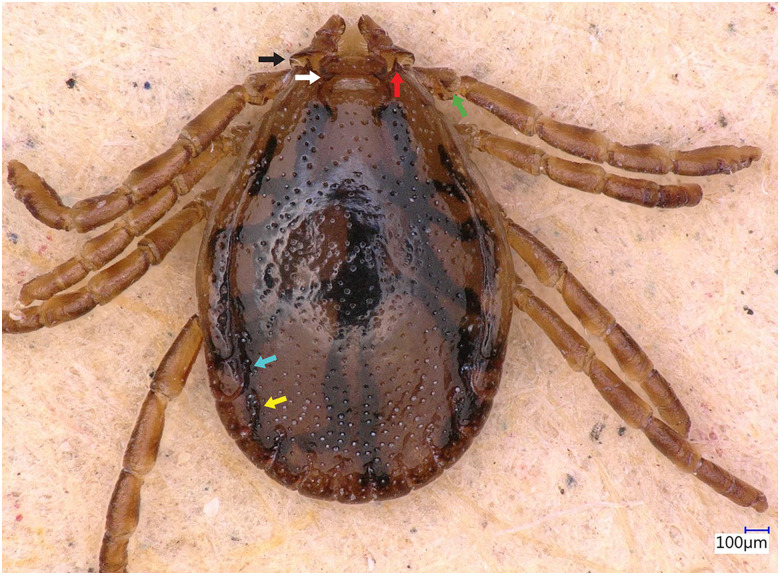
*Haemaphysalis eleonorae* male dorsal view: note the broadly salient palp article 2 (black arrow) and the broadly triangular cornua (white arrow). Coxa I anterior spurs are visible on the dorsal side (red arrow), trochanters with a small triangular spur (green arrow), lateral groove encloses first festoon at each side (cyan arrow); however, the second festoon is nearly enclosed by a groove, which does not come in contact with the lateral groove (yellow arrow).

**Figure 2.**
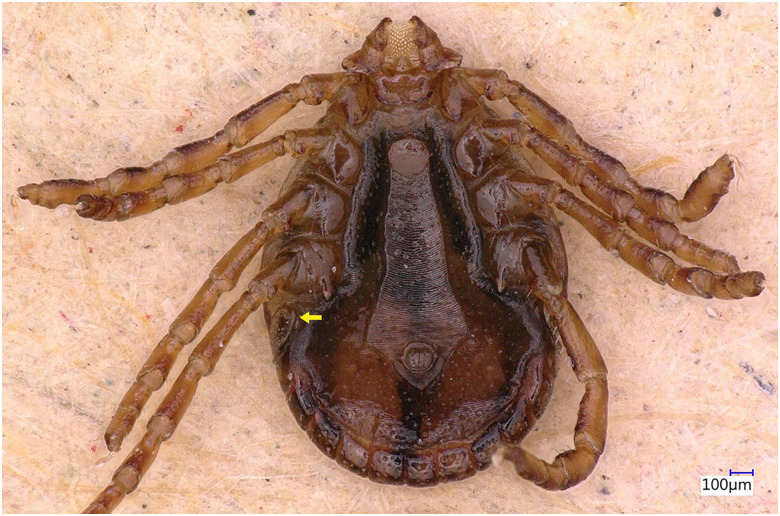
*Haemaphysalis eleonorae* male ventral view: stigmas are oval-elongated and rounded macula located on the antero-inferior side (yellow arrow).

**Figure 3.**
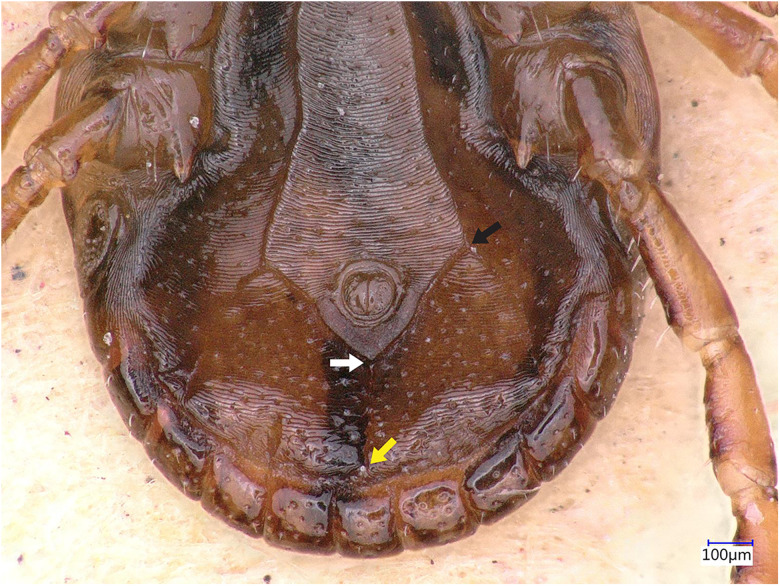
*Haemaphysalis eleonorae* male ventral view: spiracles, anus, anal groove and festoons. Note the ‘Y’ anal groove (white arrow), with ‘Y’ tail reaching the marginal groove at the level of the central festoon (yellow arrow) whereas the lateral arms diverge below the middle of the anus laterally and come together with the genital groove (black arrow).

**Figure 4.**
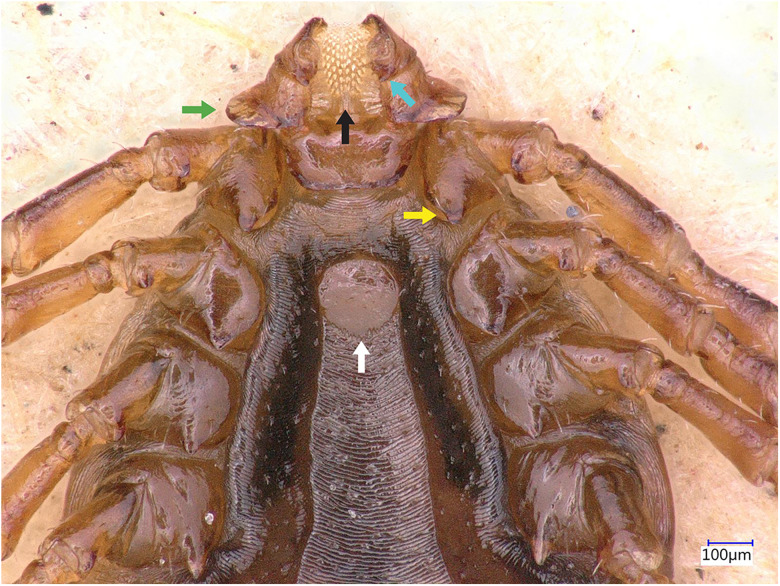
*Haemaphysalis eleonorae* male ventral view: gnathosoma, coxae and genital apron. Note the broadly salient palpal segment 2 (green arrow), the intermediary denticles of this specimen presenting a variation in the commonly observed 4/4 dental formula (black arrow), the long spur, with rounded apex on coxa I (yellow arrow), the genital apron located between coxae II, with numerous small denticles on posterior margin (white arrow) and the short palp article 3 ventral spur, which is posterointernally directed, reaching or slightly extending beyond intersegmental suture (cyan arrow).

Idiosoma: Ornamentation is indistinct on the conscutum, with reddish brown to dark reddish colour. Overall body length (from 2 slightly engorged males) is 1.76–1.85 mm (from the middle of idiosoma to the edge of the idiosoma), width 1.29–1.38 mm, ovoid, widest at the 4th coxa (Fig. 1). Punctations are shallow, moderately sized, distributed slightly dense and uniform (Fig. 1); lateral grooves distinct, beginning at the level between coxae II and III and enclosing the first of the 11 festoons at each side (Fig. 1), the second festoon is nearly enclosed by a groove, which however does not come in contact with the lateral groove (Fig. 1). Scapulae are short and blunt; cervical grooves are inconspicuous, short, slightly convex externally, converging posteriorly; eyes absent; 11 festoons ranging from 0.15 to 0.21 mm in width, ventrally delimited by a marginal groove (Fig. 2). Stigmas are oval-elongated and rounded macula located on the antero-inferior side (Figs 2 and 3); anus with ‘Y’ anal groove, ‘Y’ tail reaching the marginal groove at the level of the central festoon and the lateral arms diverge below the middle of the anus laterally and come together with the genital groove (Figs 2 and 3). Genital apron medial to coxa II, with numerous small, distinct denticles on posterior margin (Fig. 4).

Gnathosoma: Length from apices to posterior margin of the basis 0.26 mm; the basis capituli is rectangular (0.29 length, 0.07–0.08 width), 4.1 times wider than long, with the posterior margin being straight and cornua being broadly triangular (Fig. 1). The distal end of article 2 is noticeably wider than the third article, without spurs dorsally (Fig. 1). Article 3, ventral spur short, slightly curved, reaches or slightly extends beyond intersegmental suture (Fig. 4); article 4 is in the apical pit, visible ventrally; hypostome is slightly shorter than the palpi, having length 0.20–0.21 mm; dental formula is 4/4, occasionally with some irregular rudimental denticles in the middle, with 8 large teeth in the principal files, distally a rosette is visible (Fig. 4).

Legs are short and robust (Figs 1 and 2). Coxa I with long spur, with rounded apex, directed posteriorly; coxae II–III with short and moderately broad spur and rounded apex; coxa IV with a long and moderately broad spur, with narrowly rounded apex (Fig. 4). Coxa I anterior spurs are visible on the dorsal side, between scapulas and second palp segment. All trochanters have a small triangular spur (Fig. 1). Tarsus I tapers distally, measuring a length of 0.37 mm, small oval area on the dorsum of tarsus I, Haller's organ.

Chaetotaxy: Small and tiny hairs can be observed on the palps and legs. Palpal segments II have 3 hairs on the dorsal side, and 8 setae on ventral side each. Coxae have 4 setae, 1 on the dorsal point, 1 on the internal side and 2 on the external side of the spurs.

### Description of female

**Figure 5.**
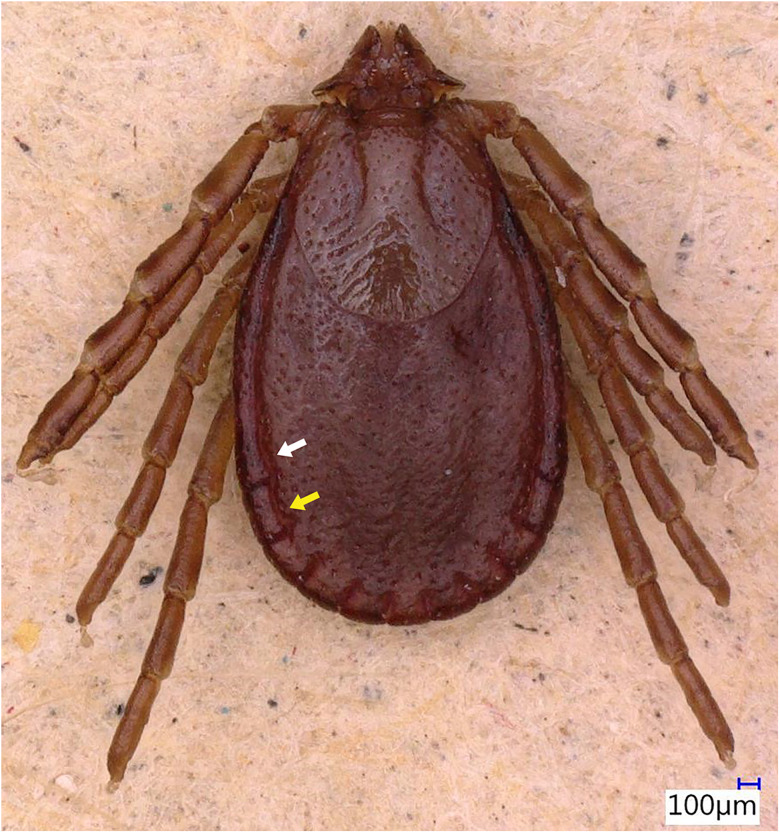
*Haemaphysalis eleonorae* female dorsal view. Note that the first 2 festoons are enclosed by the lateral grooves (yellow arrow). The first festoon is not clearly delimited (white arrow).

**Figure 6.**
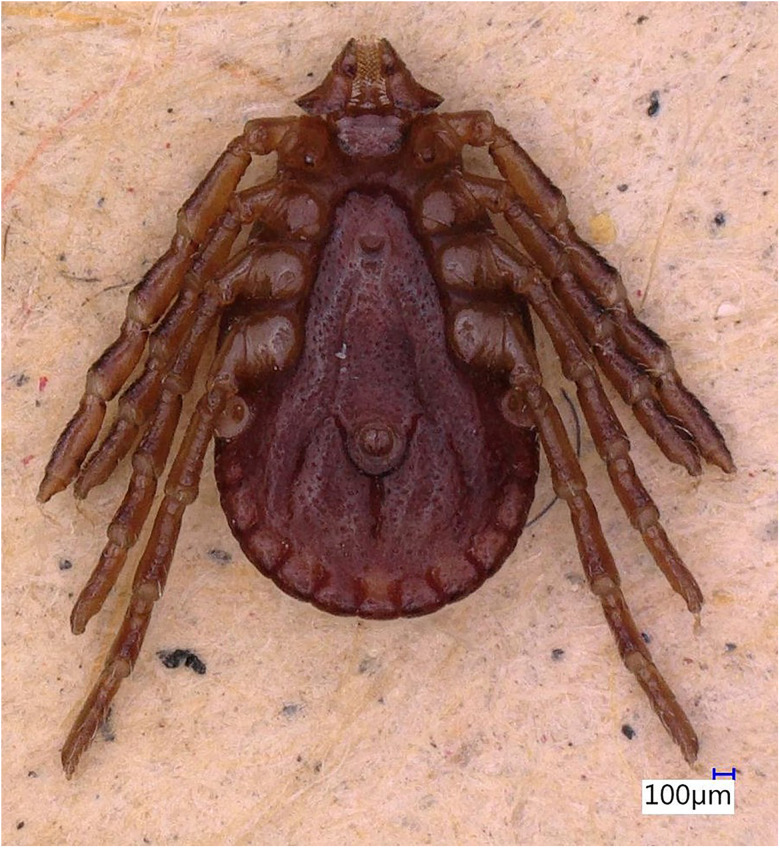
*Haemaphysalis eleonorae* female ventral view.

**Figure 7.**
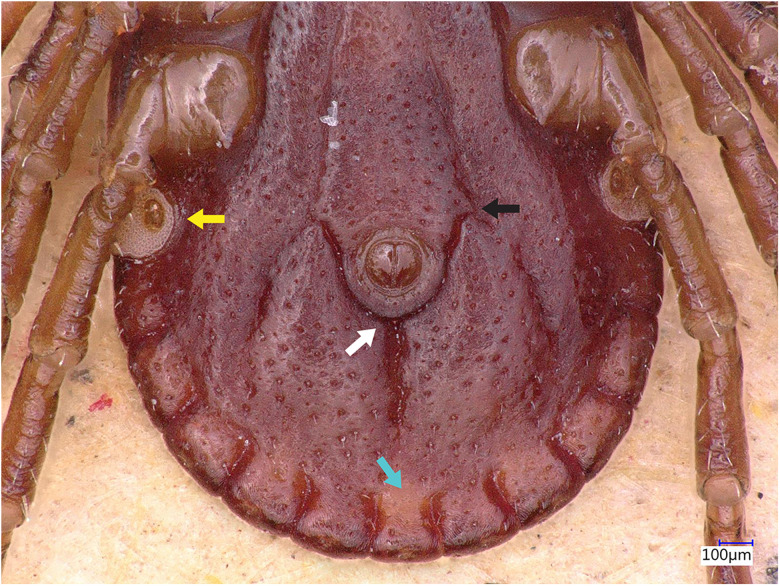
*Haemaphysalis eleonorae* female ventral view: anus, anal groove, festoons and spiracles. Stigmas are oval-elongated and rounded macula located on the antero-inferior side (yellow arrow), anus with ‘Y’ anal groove (white arrow), ‘Y’ tail not reaching the central festoon (cyan arrow) and the lateral arms diverge laterally and come together with the genital groove (black arrow).

**Figure 8.**
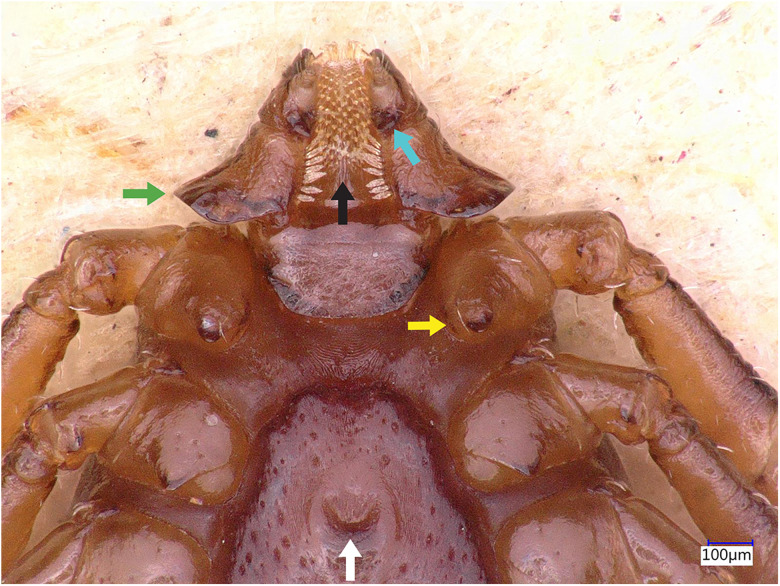
*Haemaphysalis eleonorae* female ventral view: gnathosoma, coxae and genital aperture. Note the intermediary denticles of this specimen presenting a variation in the commonly observed 4/4 dental formula (black arrow), the broadly salient palpal segment 2 (green arrow), the short palp article 3 ventral spur, which is posterointernally directed, reaching or slightly extending beyond intersegmental suture (cyan arrow), coxa I with moderately long spur and broadly rounded apex (yellow arrow) and genital aperture lips forming a broad V (white arrow).

Idiosoma: Ornamentation is indistinct on the scutum, with reddish brown to dark reddish colour. Overall body length (from 3 females; 2 unengorged and 1 slightly engorged) is 1.87–2.51 mm (from the middle to the edge of the idiosoma), width 1.16–1.53 mm, ovoid, widest at the 4th coxa (Fig. 5). Scutum is wide-oval or almost circular (length 0.74–0.99 mm, width 0.73–1.01 mm), punctations are shallow, moderately sized, distributed slightly dense and uniform (Fig. 5); scapulae are short and blunt; cervical grooves are inconspicuous, long, almost parallel, slightly divergent anteriorly and posteriorly, eyes absent; lateral grooves are evident, beginning at the level between coxa II, from the scutum down enclosing the first 2 festoons at each side. Eleven festoons ranging from 0.15 to 0.26 mm in width, without ventral marginal groove, first festoon is not well delimited on the dorsal side (Fig. 5). Stigmas are oval-elongated and rounded macula located on the antero-inferior side (Figs 6 and 7); anus with ‘Y’ anal groove, ‘Y’ tail not reaching the central festoon and the lateral arms diverge laterally and come together with the genital groove (Fig. 7). The genital aperture is located between the coxae II and III, genital aperture lips form a wide ‘V’ (Fig. 8).

**Figure 9.**
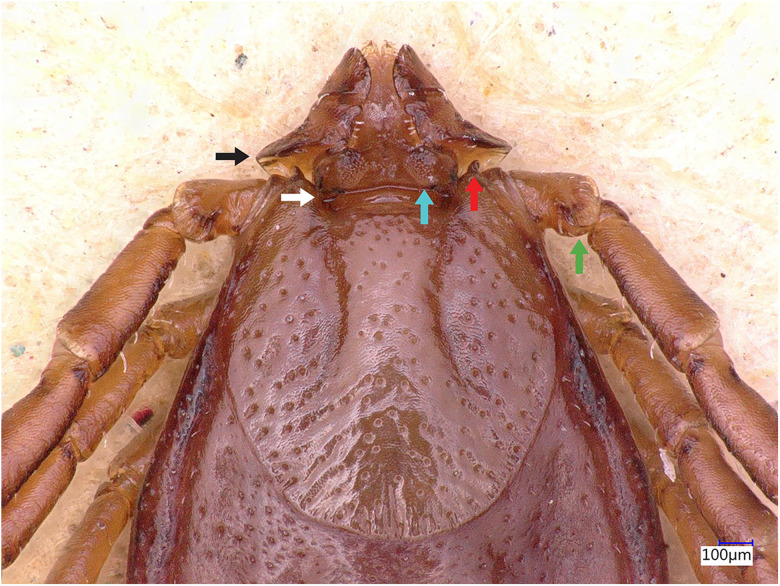
*Haemaphysalis eleonorae* female dorsal view: scutum and gnathosoma. Palp article 2 is broadly salient (black arrow), cornua is broadly triangular (white arrow), distance between oval porose areas large, they nearly reach the posterior margin of the basis capitula (cyan arrow). Coxa I anterior spurs visible dorsally (red arrow). Trochanters with a small triangular spur (green arrow).

Gnathosoma: Length from apices to posterior margin of the basis 0.29–0.44 mm; the basis capituli is rectangular (length 0.09–0.12, width 0.37–0.43), 3.5–4.1 times wider than long, with the posterior margin being straight, and cornua are broadly triangular (Fig. 9). Porose areas oval, length 0.09–0.10 mm, wide 0.09–0.12 mm with large distance between them, almost touching the posterior margin. Palpi are broad. The distal end of article 2 is noticeably wider than the third article. Article 3, ventral spur short, slightly curved, extents slightly beyond intersegmental suture; article 4 is in the apical pit, visible ventrally; hypostome is slightly shorter than the palpi, having length 0.30–0.33 mm; dental formula is 4/4, with 8 teeth in the principal files, distally a rosette is visible (Fig. 8).

Legs are short and robust (Figs 5 and 6). Coxa I with moderately long spur, with broadly rounded apex, directed posteriorly; coxa II with short spur, with broadly rounded apex; coxae III–IV with longer spur, with a narrowly rounded apex (Figs 6 and 8). Coxa I anterior spurs are visible on the dorsal side, between scapulas and second palps segment (Fig. 9). All trochanters have a small triangular spur. Tarsus I tapers distally, measuring a length of 0.47–0.59 mm, oval small area on the dorsum of tarsus I, Haller's organ.

Chaetotaxy: Small and tiny hairs can be observed on the palps, legs and idiosoma both dorsally and ventrally. Palpal segments II have 3 hairs on the dorsal side, and 6 setae on ventral side each.

### Description of nymph

**Figure 10.**
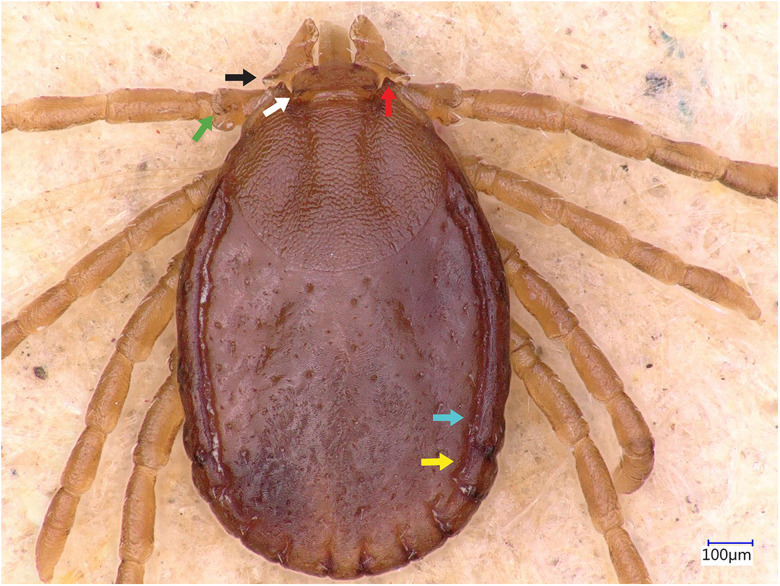
*Haemaphysalis eleonorae* nymph, dorsal view. Note that the first 2 festoons are enclosed by the lateral grooves (yellow arrow). The first festoon is not clearly delimited (cyan arrow). Palpal segment 2 is broad (black arrow), cornua broadly triangular (white arrow), coxa I anterior spurs visible dorsally (red arrow) and trochanter with a small spur (green arrow).

**Figure 11.**
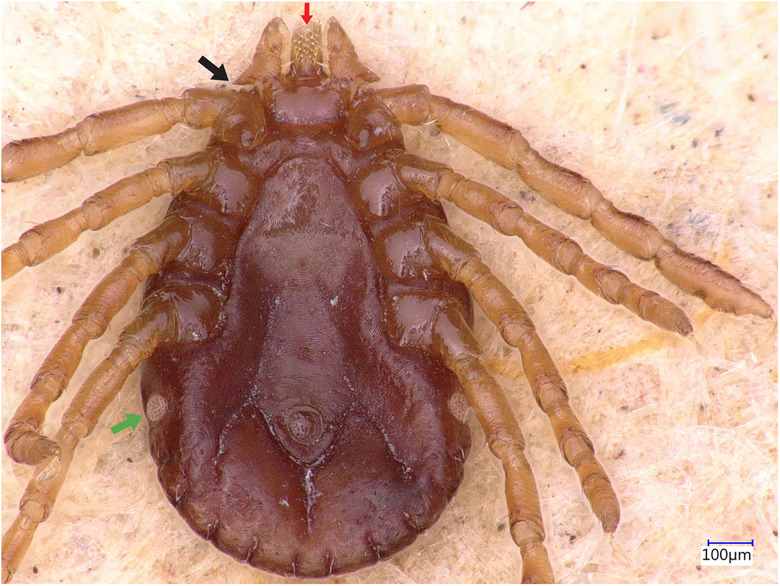
*Haemaphysalis eleonorae* nymph, ventral view. Palp articles 2 are broad (black arrow) and dental formula presents with a 3/3 pattern (red arrow).

Idiosoma: Ornamentation is indistinct on the scutum, with reddish brown colour. Overall body length (from 3 nymphs) is 0.85–1.09 mm (from the middle to the edge of the idiosoma), width 0.61–0.69 mm, ovoid, widest at the 4th coxa (Fig. 10). Scutum is wide-circular (length 0.31–0.39 mm, width 0.40–0.50 mm), punctations absent, presence of rugose transversal depressions (Fig. 10); scapulae blunt; cervical grooves, long, almost parallel, slightly divergent at the end, not reaching the scutum edge; eyes absent; lateral grooves are evident, extending from the scutum down enclosing the first 2 festoons at each side. Eleven festoons ranging from 0.06 to 0.11 mm in width, first festoons are not well delimited dorsally, without ventral marginal groove (Figs 10 and 11). Stigmas are almost round (0.05–0.06 mm) (Fig. 11); anus with ‘Y’ anal groove, ‘Y’ tail not reaching the central festoon and the lateral arms diverged laterally and come together with the genital groove (Fig. 11).

Gnathosoma: Length from apices to posterior margin of the basis 0.14–0.15 mm; the basis capituli is rectangular (length 0.04–0.05, width 0.17–0.19), 3.8–4.2 times wider than long, with the posterior margin being straight and cornua are broadly triangular (Fig. 10). Palpi are broad. Hypostome is slightly shorter than the palpi, having length 0.10–0.11 mm; dental formula is 3/3, with 4–5 teeth in the principal files, distally a rosette is visible (Fig. 11).

Legs are short and robust (Figs 10 and 11). Coxae with moderately long spur, with broadly rounded apex, directed posteriorly. Coxa I anterior spurs are visible on the dorsal side, between scapulas and second palps segment (Fig. 10). Tarsus I measuring a length of 0.25–0.29 mm, oval small area on the dorsum of tarsus I, Haller's organ.

Chaetotaxy: Small and tiny hairs can be observed on the palps, legs and idiosoma dorsal and ventral. Palpal segments II have 1 seta on the dorsal side, and 3 setae on ventral side each.

### Remarks

*Haemaphysalis* is divided into 3 groups based on morphology: primitive, intermediate and structurally advanced. The structurally advanced group contains 6 *Haemaphysalis* subgenera, namely *Ornithophysalis*, *Haemaphysalis*, *Kaiseriana*, *Aborphysalis*, *Segalia* and *Rhipistoma* (Geevarghese and Mishra, 2011).

The new species is based on its morphological features: 11 festoons, basis capituli rectangular without any lateral expansion in both immature and adults, dental formula 3/3 for nymphs and 4/4 for adults, belongs to the structurally advanced (Geevarghese and Mishra, 2011).

The collected ticks lack any pronounced palpal segment 2 spurs and/or spur-like angles that characterize all adults and nymphs and most larvae of *Rhipistoma*. Therefore, *Rhipistoma* can be ruled out. In addition, palp article 3 ventral spur is short (reaching or slightly exceeding the border between articles 2 and 3), so it belongs to the *Ornithophysalis* subgenus. The species belonging to this subgenus seem to have abruptly evolved from structurally primitive. Six species from the known 20 parasitize only birds (Geevarghese and Mishra, 2011). The adults have a 4/4 dental formula as *H. hoodi*, while some showed some intermediary denticles like a 5/5 dental formula as *H. phasiana*. However, according to Saito *et al*. (1974), this is the most important character to distinguish *H. phasiana* from the *H. doenitzi* group, which comprises 4 mainly ornithophilic tick species (*H. phasiana*, *H. hoodi*, *H. madagascariensis*, *H. doenitzi*). In female of *H. eleonorae* the first festoons are not well delimited in the posterior part as in *H. hoodi*.

Nymph has 3/3 dental formula.

### Comparison of ticks belonging to the *H. doenitzi* group

Table 1 provides the critical characters for morphological differentiation of the ticks belonging to the *H. doenitzi* group based on previous literature (Warburton and Nuttall, 1909; Hoogstraal, 1966; Hoogstraal and Wassef, 1973; Saito *et al*., 1974; Horak *et al*., 2018).

**Table 1.** Morphological comparison of *Haemaphysalis doenitzi* group species adults with *Haemaphysalis eleonorae* sp. nov. based on Warburton and Nuttall (1909), Hoogstraal (1966), Hoogstraal and Wassef (1973), Saito *et al*. (1974) and Horak *et al*. (2018)

Morphological characteristics	*H. doenitzi*	*Haemaphysalis phasiana*	*Haemaphysalis madagascariensis*	*Haemaphysalis hoodi*	*H. eleonorae* sp. nov.
Dental formula	4/4	5/5 (anterior or posterior rows may be 6/6 or 7/7)	4/4	4/4	4/4
Palpal segment 3 ventral spur	Male/female: broad/reaching intersegmental suture, posteriorly or posterointernally directed	Male: ventral spur posterointernally directed, broad anteriorly, tapering to a sharp apex projecting internally at level of intersegmental suture Female: not mentioned	Male: ventral spur of segment 3 medially directed, bluntly rounded apically, and not reaching internal or posterior margin of segment Female: more narrowly rounded and slightly overlapping internal margin of segment	Male: ventral spur short, narrow, triangular, projecting slightly beyond juncture with palpal segment 2, usually medially directed Female: ventral spur short, narrow, triangular, with sharp tip reaching slightly beyond juncture of segments 2 and 3	Male/female: Article 3, ventral spur short, posterointernally curved, reaches or slightly extends beyond intersegmental suture
Ventrointernal setae	Male: 8 Female: 7 (some variation observed depending on material)	Male: ca. 8 Female: numbering more than 8 (mostly 10–12)	Male: 4 Female: 5	Not mentioned in descriptions	Males: varying between 3 and 7, most commonly between 4 and 6 Females: varying between 4 and 8, mostly between 6 and 8
Festoons	Male: 2 festoons enclosed Females: 2 festoons enclosed	Male: 2 festoons enclosed (rarely 1 festoon) Female: 2 festoons enclosed	Male: 1 festoon enclosed Female: not mentioned (probably because specimens greatly engorged)	Male: 1 festoon enclosed Female: 2 festoons enclosed based on figure in Paguem *et al*. (2022), not mentioned how many festoons enclosed in initial description by Warburton and Nuttall (1909), in Horak *et al*. (2018) only the first festoon is depicted as enclosed	Male: 1 festoon enclosed, notably, the second festoon is nearly enclosed by a groove, which however does not come in contact with the lateral groove Female: 2 festoons enclosed
Cornua	Cornua triangular, ca. one-third (male) or one-fourth (female) as long as base of basis capituli	Cornua triangular, ca. one-third (male) or one-fourth (female) as long as base of basis capituli	Cornua widely triangular, one-fourth (male) or one-third (female) as long as base	Male: small but distinct cornua Female: very small cornua	Male/female: cornua broadly triangular
Scutum	Male: punctations moderately numerous, mostly small, shallow, rather regularly distributed, somewhat more numerous and often slightly larger anteriorly Female: punctations as in male anteriorly, or moderately large, deeper, and more rugose, especially when fed	Male: punctations moderately numerous, size mostly moderate, some small, irregularly distributed Female: punctations about as in male anteriorly	Male: punctations widely spaced, small, shallow (initially described as numerous) Female: punctations spaced as in male but mixed medium size and large	Male: scutum is beset with a moderate number of fairly large, shallow punctations Female: scutum is broadly oval, slightly longer than wide and gradually converging posteriorly, large evenly scattered punctations	Male/female: punctations are shallow, moderately sized, distributed slightly dense and uniform
Coxa spurs	Male: coxae each with a short, broad spur; I and IV spurs usually subequal (I sometimes smaller than IV), broadly triangular, extending somewhat beyond posterior margin of coxa; II and III spurs more broadly rounded, shorter; IV spur often posterointernally directed Female: coxal spurs variable in size	Coxae each with a broadly triangular spur; I and II spurs subequal, extending somewhat beyond coxal posterior margin; III spur equally as long or shorter; IV spur larger than others, posterointernally directed. In females, coxa spurs somewhat larger and often more pointed apically	Male/female: each coxa with a short, wide triangular spur extending slightly beyond posterior margin	Male: coxa I with a short triangular posteromedial spur; coxae II and III each with a short, broad, triangular posterocentral spur; coxa IV with a narrow sharp-tipped triangular posteromedial spur Female: coxae I and IV each with a triangular posteromedial spur; coxae II and III each with a short broadly arcuate posterocentral spur scarcely protruding beyond coxal margin	Male: coxa I with long spur, with rounded apex, directed posteriorly, coxae II–III with short and moderately broad spur and rounded apex, coxa IV with a long and moderately broad spur, with narrowly rounded apex Female: coxa I with moderately long spur, with broadly rounded apex, directed posteriorly; coxa II with short spur, with broadly rounded apex, coxae III–IV with longer spur, with a narrowly rounded apex
Lateral grooves	Male: long/broad/deep	Male: long/broad/deep	Male: long	Male: well-marked, of medium length	Male: distinct, beginning at the level between coxae II and III
Comparison between species considering notes in the manuscripts		Among the adults of this group, *H. phasiana* is the only species with a 5/5 dental formula and with female ventrointernal setae numbering more than 8. Among the nymphs, the dental formula is 2/2 in each other species but most often 3/3 in *H. phasiana*	*Haemaphysalis* (*R.*) *madagascariensis* is distinguished from *H*. (*R.*) *doenitzi* by very slight differences in palpal outlines and small size of the spur on coxa IV. Notably, the basal width of palpal segment 3 in the Asiatic species is slightly wider than the apical width of 2; in the Madagascar species these widths are equal. The ventral integument of the male *H. madagascariensis* is much more punctate than that of *H. doenitzi*		Female of *H. eleonorae* sp. nov. can be distinguished from *H. hoodi* by the broadly triangular cornua in the former, while it is small in the latter. Males of the new species can be differentiated from *H. hoodi* as in females. In addition, the second festoon in *H. hoodi* is not reported to be partially enclosed by the groove as in *H. eleonorae* sp. nov. Nymphs of *H. eleonorae* sp. nov. possess a 3/3 dental formula as opposed to *H. hoodi*, which demonstrates a 2/2 dental formula

*Note:* Key characteristics are detailed in the referenced literature, in addition to the figures provided herein.

### Phylogenetic analysis

**Figure 12.**
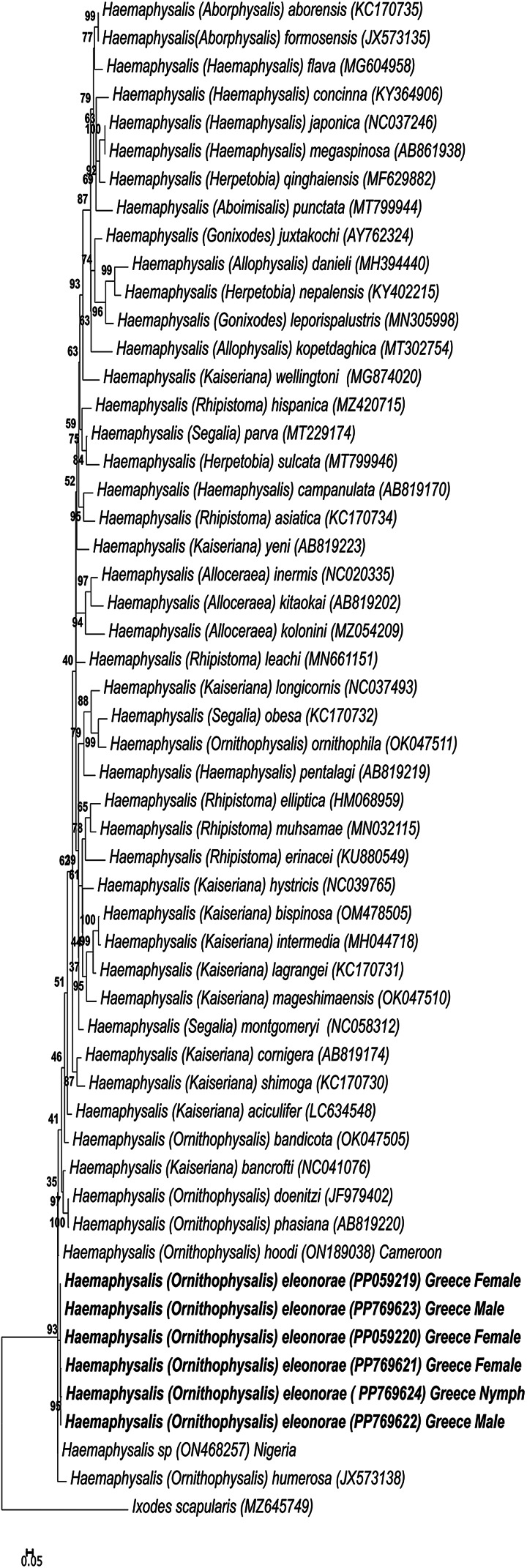
Phylogenetic analysis of the 16S rRNA gene. Indicated are species names and subgenera followed by their GenBank accession number. The sequences from the current study are indicated in bold. Bootstrap values above 80% are indicated.

**Figure 13.**
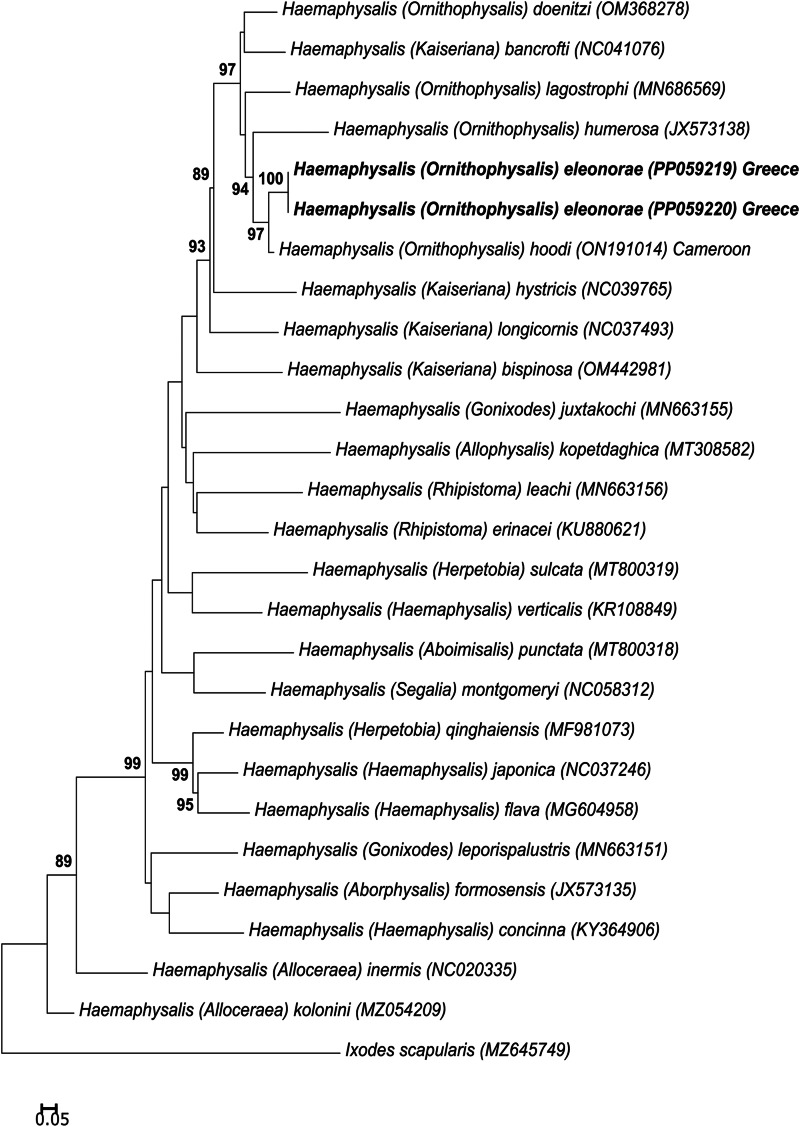
Phylogenetic analysis of the COI gene. Indicated are species names and subgenera followed by their GenBank accession number. The sequences from the current study are indicated in bold. Bootstrap values above 80% are indicated.

**Figure 14.**
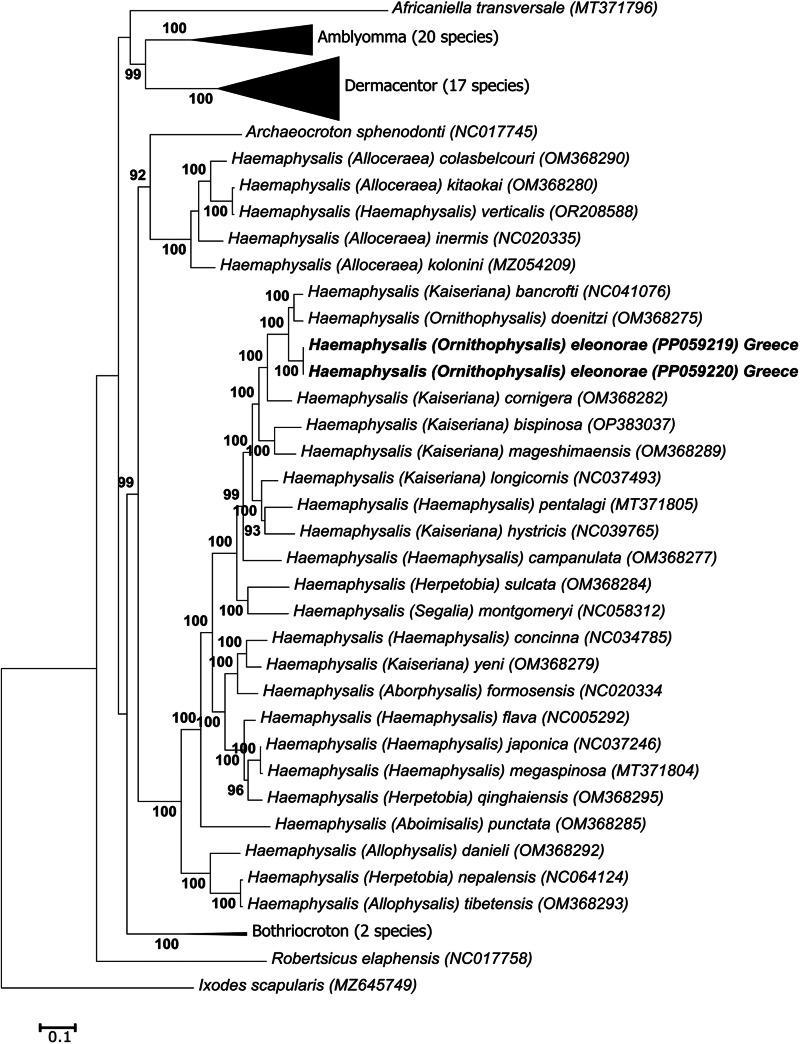
Phylogenetic analysis of the mitochondrial protein genes. Indicated are species names and subgenera followed by their GenBank accession number. The sequences from the current study are indicated in bold. Bootstrap values above 80% are indicated. Genera with more than 1 species have been collapsed and number of species in clade indicated.

Phylogenetic analysis using the 16S gene indicates that *H*. *eleonorae* groups with *H. hoodi* from Cameroon as well as a sequence from Nigeria and *Haemaphysalis humerosa* forming a well-supported clade (Fig. 12). It also indicated that all 16S gene sequences from females, males and the nymph of the new species grouped in the same clade and showed >99% sequence identity. This clade is in turn nested within another well-supported clade with other members of *Ornithophysalis* and *Kaiseriana*. Similarly, for the COI gene, *H. eleonorae* groups with *H. hoodi* from Cameroon in a clade composed of other members of the *Ornithophysalis* and *Kaiseriana* (Fig. 13). The same grouping is observed for the analysis based on all mitochondrial proteins (Fig. 14). No strong support is observed for the monophyly of any of the subgenera.

## Discussion

Migratory birds are well known to transport various tick species over long distances, climate zones and continents. A number of ticks, infesting other long-distance migratory birds (passerines and near-passerines species) have been detected in Antikythira, with the most abundant tick species belonging to *H. marginatum* and *H. rufipes* (Wallménius *et al*., 2014; Hoffman *et al*., 2021). The detection of a new tick species which to the current knowledge seems to be associated specifically with Eleonora's falcons, in a single breeding island of the species so far, is therefore important. However, identifying possible areas of exposure to this tick species and/or possible modes of dispersal based on the information in hand is challenging considering that Eleonora's falcon is well-known for unique ecological adaptations that introduce further complexity.

Eleonora's falcons are aerial hunters, feeding almost exclusively on flying insects, except for the breeding period during which they rely on migratory birds (Walter, 1979; Ristow, 2004; Xirouchakis *et al*., 2019). In fact, the breeding period (mid-July–late October) has been fine-tuned with the passage of autumn migratory birds, an abundant food source intercepting the rocky islands where Eleonora's falcons breed colonially (Walter, 1979; Xirouchakis *et al*., 2019). For its increased insularity the Aegean Sea hosts more than 80% of the species' global breeding population (Dimalexis *et al*., 2008) that comprises of 29 200–29 600 mature individuals (BirdLife International, 2023). Following a trans-equatorial voyage of more than 7000 km, the falcons spend approximately 5 months in the Malagasy region visiting mainly rainforests and cultivated areas (Kassara *et al*., 2017; Hadjikyriakou *et al*., 2020). Compared to a less consistent stopover pattern in autumn, on their way back to the Mediterranean in spring, they refuel intensively in savannas and shrublands in the Horn of Africa (Gschweng *et al*., 2008; Kassara *et al*., 2014; Vansteelant *et al*., 2023 and references therein). Upon reaching their breeding grounds and until the onset of the breeding period, Eleonora's falcons can be observed in a variety of habitats across the Mediterranean region [e.g. forests, shrublands, cultivated areas, rivers (Kassara *et al*., 2019)], up to several hundred kilometres away from their breeding colonies during the pre-breeding period from April to July (Ristow and Wink, 1992–1994; Gschweng *et al*., 2008; Ristow, 2010; Mellone *et al*., 2013; Kassara *et al*., 2022). Given the extensive range and diverse habitats frequented by Eleonora's falcons throughout their annual cycle, understanding the potential pathways for the spread of *H. eleonorae* is intricate. Considering the infestation of both adults and nestlings during the breeding period, months after their arrival in the Mediterranean, we can at least safely assume that this tick species is established on the island of Antikythira. Future studies should explore the distribution of this tick species in other breeding colonies across its range, including those in the Aegean Sea. Additionally, data on Eleonora's falcons' infestation with ticks from the Malagasy region and sub-Saharan Africa would be necessary; however, their collection might be hindered by increased logistical and field-related constraints.

Reports on the established populations of tick species belonging to the *H. doenitzi* group seem to be missing from the Western Palaearctic, whereas the status of *H. pavlovskyi* is uncertain (Sames *et al*., 2008). *Haemaphysalis phasiana*, which is assumed to be present in the Palaearctic was so far only described for its central/eastern part (Japan, Korea, Eastern Russia, Turkmenistan and China), whereas *H. hoodi* rather seems to have a sub-Saharan distribution (Hoogstraal and Wassef, 1973; Sames *et al*., 2008; Paguem *et al*., 2022). A single report on *H. hoodi* parasitizing *Falco tinnunculus* from Morocco and a *Haemaphysalis* sp. on Eleonora's falcons from Algeria has been additionally identified within the existing scientific literature (Bailly-Choumara *et al*., 1980; Laid *et al*., 2019). *Haemaphysalis madagascariensis* was so far only described from Madagascar (Hoogstraal, 1966), whereas *H. doenitzi* seems to have an Oriental–Australian distribution (Hoogstraal and Wassef, 1973). The presence of the former species also needs careful re-evaluation according to Guglielmone *et al*. (2014). Falconidae parasitism by *H. doenitzi* has been reported according to Guglielmone *et al*. (2014) and *H. hoodi* in the past (Hoogstraal, 1956). Our findings emphasize the probable significance of Falconidae as substantial hosts for tick species within the *H. doenitzi* group, underscoring the necessity for additional investigation into their impact on the ecology of these ticks.

Future investigations into the newly described species should prioritize an in-depth examination of its morphological and phylogenetic connections with closely related species within the *H. doenitzi* group, given the still limited mitochondrial genome data availability within the *Ornithophysalis* subgenus. Additionally, emphasis should be placed on exploring its ecology, biology and epidemiological importance, bearing relevance to both Falconidae conservation efforts and implications for public health. Considering the recent phylogenetic study on *H. hoodi* (Paguem *et al*., 2022), the findings of the current study seem to be timely, also considering Saito *et al*.'s (1974) comments on the necessity for studies from a greater number of regions and locations.

## Data Availability

All data generated or analysed during this study are included in this published article. Generated sequences were submitted to GenBank as stated in the main text.
